# Lauryl-poly-L-lysine: A New Antimicrobial Agent?

**DOI:** 10.1155/2014/672367

**Published:** 2014-02-23

**Authors:** Laetitia Vidal, Véronique Thuault, Arturo Mangas, Rafael Coveñas, Anne Thienpont, Michel Geffard

**Affiliations:** ^1^Institut pour le Développement de la Recherche en Pathologie Humaine et Thérapeutique (IDRPHT), 33400 Talence, France; ^2^Laboratoire Biogam, 33600 Pessac, France; ^3^Laboratory of Neuroanatomy of the Peptidergic Systems (Lab. 14), Institute of Neurosciences of Castilla y León (INCYL), 37007 Salamanca, Spain; ^4^Institut des Sciences Moléculaires (ISM) à ENSCPB, 33600 Pessac, France; ^5^Laboratoire de l'Intégration du Matériau au Système (IMS) à l'Ecole Nationale Supérieure de Chimie et de Physique de Bordeaux (ENSCPB), 33600 Pessac, France; ^6^IDRPHT, 200 Avenue Thouars, 33400 Talence, France

## Abstract

The development of multiple antibiotic resistance is a global problem. It is necessary to find new tools whose mechanisms of action differ from those of currently used antibiotics. It is known that fatty acids and cationic polypeptides are able to fight bacteria. Here, we describe the synthesis of fatty acids linked to a polypeptide with antibacterial activity. The linkage of fatty acids to a polypeptide is reported to increase the antibacterial effect of the linked fatty acid in comparison with free fatty acids (FA) or free poly-L-lysine (PLL) or a mixture of both (FA free + PLL free). A number of C_6_–C_18_ fatty acids were linked to PLL to obtain new synthetic products. These compounds were assessed *in vitro* to evaluate their antibacterial activity. Some fatty acid-PLLs showed a good ability to fight bacteria. Their bactericidal activity was evaluated, and, lauryl linked to PLL was found to be the most active product against both Gram-positive and Gram-negative bacteria. This new active component showed a good degree of specificity and reproducibility and its minimum inhibitory concentration (MIC) was comparatively good. The antibacterial activity of the lauryl-PLL compound suggests that it is a new and promising antimicrobial agent.

## 1. Introduction

The rapid emergence of multiresistant bacteria is a major global health problem. During the last 10 years, one goal of the scientific community has been to generate surveillance systems focused on antimicrobial resistance. To date, health services are continuously adapting their functions according to geographical situation and life styles of the populations [1].

In the United States, in medical facilities and the general community, more deaths have been caused by methicillin-resistant *Staphylococcus aureus* (MRSA) infections than by HIV/AIDS [2]. In the case of many strains of *S. aureus*, with the exception of glycoproteins, all antimicrobial agents tested were found to be ineffective, including second- or third-generation antibiotics [3]. More resistant strains of common pathogenic germs are now appearing and are spreading faster than before. Thus, it is crucial to find new products with different mechanisms of action other than the antibiotics available on the market.

Over the past few years, many research groups have focused their attention on the search for new therapeutic approaches for the treatment of infectious diseases. In this sense, it is known that antimicrobial peptides (AMPs) exert broad-spectrum activity against bacteria, viruses, and fungi. These cationic peptides have been defined as having less than 50 amino acid residues. AMPs are generally characterized by the presence of many alanine, arginine, and lysine residues and a substantial portion (above or equal to 30%) of hydrophobic residues [4, 5]. Amphipathicity, the innate immune response, and the interaction of a hydrophobic interface with the bacterial membrane are their main characteristics [6].

Other promising candidates for biomedical and pharmaceutical applications are the homo-poly-amino acids, an important class of biodegradable polymers. Among them, poly-L-lysine (PLL) and its derivatives have been studied most extensively. They are water-soluble, edible, and nontoxic with respect to the environment and humans [7]. In general, cationic polypeptides (such as *α*-PLL) have been used as auxiliary agents in drug delivery systems. Cationic polypeptides can be responsible for membrane lysis activity or a signal of nuclear location [8]. *α*-Poly-D-Lysine, a structure close to the PLL polypeptide, obtained by chemical synthesis was used as a carrier for methotrexate by Ryser and Shen [9]. This conjugation increased cellular uptake and improved drug transport by reducing drug resistance [9, 10].

An innovative therapy devoid of side effects, Endotherapia [11–15], has used PLL derivatives. *α*-PLL and fatty acids are known to have an antimicrobial effect. *In vitro*, several fatty acids at a concentration higher than 1 mg/mL show a broad spectrum of antimicrobial activity against a large variety of bacteria. Lauric acid (C_12_) was found to have the highest antimicrobial activity against Gram-negative organisms [16]. Although free fatty acids are not as effective as antimicrobial agents, they may be used as potential antimicrobial products and as a preservative for ointments. Regarding C_6_–C_18_ fatty acids, their activities have been described to be specifically directed against Gram-positive bacteria [17] and only a few Gram-negative strains have antimicrobial susceptibility to short-chain fatty acids [18].

In the present paper, we describe the *in vitro* activities of synthetic fatty acid-PLL compounds against bacterial strains. These compounds result from the linkage of fatty acids to PLL polypeptide thanks to previous methodology applied in work in Endotherapia. Here, we describe the characterization of a fatty acid, lauric acid covalently attached to PLL, that exerts reproducible antibacterial activity against both Gram-positive and Gram-negative bacteria. This work opens new perspectives in anti-infective therapeutic strategies.

## 2. Material and Methods

### 2.1. Bacterial Strains and Culture

In the present study, the following bacterial strains, multiresistant or not, were obtained from clinical isolates: (a) Gram-positive strains: the *Staphylococcaceae* (*S*. *aureus*,* S. epidermidis*), *Streptococcaceae *(*pneumoniae*,* A*,* B*); (b) Gram-negative strains: *Enterobacteriaceae* species (*Enterobacter cloacae*,* Escherichia coli*,* Klebsiella pneumoniae*,* Klebsiella oxytoca*,* Morganella morganii*,* Proteus mirabilis*,* and Serratia marcescens*) and the *Pseudomonadaceae* (*P. aeruginosa*,* P. luteola*,* and P. putida*).

The antibiogram of Gram-positive and Gram-negative bacterial strains was obtained using the Vitek 2 compact (bioMérieux) technique according to the recommendations of the Antibiogram Committee of the French Microbiological Society (CA-SFM) and Clinical and Laboratory Standard Institute (CLSI). This is an automat (bioMérieux) of bacterial and antibiogram identification with product number GP-21,342 for Gram-positive and GN-21,341 for Gram-negative bacteria. The majority of the bacterial strains used were grown on Mueller-Hinton medium and the anaerobic bacterial strains were grown on Wilkins-Chalgren medium.

The antibacterial activity of the fatty acid-PLL compounds was determined by the diffusion method on agar seeded with the bacteria under study. The concentration of bacteria used was evaluated by densitometry. It always had a value of 0.5 on the McFarland scale, corresponding to 1.5 × 10^8^ bacteria/mL. Twenty-five microliters of each product was placed in each Petri dish previously inoculated with a bacterial strain. After twenty hours of incubation, the inhibition area was measured in order to calculate the area of bacterial lysis (cm^2^). Moreover, a series of tests were performed to evaluate bacterial kinetic activity.

### 2.2. Synthesis of Fatty Acid-PLLs

As previously reported [19, 20], fatty acids and PLL were used to synthesize fatty acid-PLLs. The saturated fatty acids used were caproic acid (C_6_, Sigma-Aldrich), caprylic acid (C_8_, Acros Organics), capric acid (C_10_, Sigma-Aldrich), lauric acid (C_12_, Acros Organics), myristic acid (C_14_, Acros Organics), and palmitic acid (C_16_, Acros Organics), whereas the unsaturated fatty acids used were palmitoleic acid (C_16:1_, Fluka), oleic acid (C_18:1_, Sigma-Aldrich), and linoleic acid (C_18:2_, Sigma-Aldrich). The cationic *α*-PLL (Alamanda Polymers; molecular mass around 21,000 Da) is a polypeptide composed of repeated structural units of 100 lysine residues and PLL was analyzed by gas chromatography and nuclear magnetic resonance for molecular mass determination and polydispersity index.

**Figure 1:**
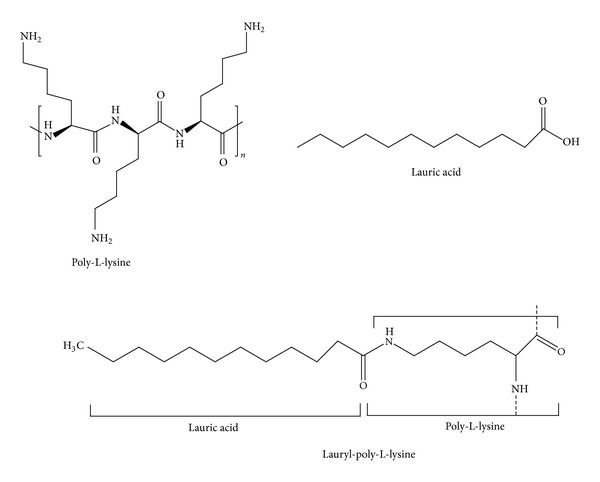
Molecular structure of poly-L-lysine, lauric acid, and lauryl-poly-L-lysine. *α*-Poly-L-lysine (*α*-PLL) was built with the lysyl residue with linkages between the *α*-carboxyl and *α*-amino groups. The lysyl pattern was repeated (*n* = 33). The lauric acid was C_12_H_24_O_2_, called dodecanoic acid. The lauric acid was linked to PLL in random fashion.

Each fatty acid was activated by ethyl chloroformate (ECF), which acts as a coupling agent by activating the carboxylic group of the fatty acids. The intermediate compound was linked to the *ε*-amine group of the lysine residue through amide-bond formation [21]. This was then purified by dialysis in buffer solutions and lyophilized for analysis. Thus, the final compounds were called fatty acid-PLLs. For example, lauric acid linked to PLL was called lauryl-PLL (see Figure 1).

### 2.3. Analytical Methods

For identification, fatty acid-PLL compounds were analyzed by Attenuated Total Reflectance-Fourier Transformed InfraRed (ATR-FTIR) spectroscopy (with a Bruker apparatus). The linkage of fatty acids to PLL was controlled by FTIR in aqueous solution [22, 23]. Circular dichroism enabled us to identify the secondary structure of fatty acid-PLLs, both in random coil form and in the *α*-helix form and in the *β*-sheet form. For quantification, the concentration of fatty acids linked to PLL was calculated by gas chromatography. After the acidolysis and the hydrolysis of fatty acid-PLL, the free fatty acids were derivatized [24] and analysed by gas chromatography (GC Varian 3,500) coupled to a flame ionization detector (FID) [25, 26]. A control solution of fatty acid was prepared and analysed under the same experimental conditions as the test solution.

### 2.4. Evaluation of Carrier Specificity

In order to evaluate carrier specificity, we linked fatty acids to other carriers under the same conditions as for the PLL compounds: poly-L-ornithine (PLO) [27], and the oligomer polyethylene glycol (PEG) [28] were used. PEG is well known in the pharmaceutical industry as a dispensing agent, an excipient for ointments and drugs, and as a carrier. The PEG (HO–CH_2_–(CH_2_–O–CH_2_–)_*n*_–CH_2_–OH) used had a molecular mass of 20,000 Da (Fluka). The PLO was made in our laboratory from L-ornithine (C_5_H_12_N_2_O_2_) (Bachem) with glutaraldehyde OHC–(CH_2_)_3_–COH, resulting in a polycondensation reaction and the formation of imines, which were reduced with sodium borohydride. We obtained poly-(L-ornithine)-G, whose apparent molecular mass was calculated to be around 20,000 Da by electrophoresis [29].

### 2.5. Determination of the Minimum Inhibitory Concentration (MIC) and Kinetic Activity of Lauryl-PLL

A study was made of the MIC and the kinetic activity of the lauryl-PLL compound. MIC determination was carried out on agar by the diffusion method, allowing us to measure the diameter of the inhibition area. The observation of the starting colony on the inhibition area defined the limit of the strongest antibacterial effect.

In the case of the *S. aureus*, *P. aeruginosa,* and *E. coli* bacterial strains, the MIC was the lowest concentration that inhibited the growth of the bacteria, showing a concentration range from 31 to 2000 *μ*M of grafted lauric acid.

Study of the inhibitory kinetics of lauryl-PLL activity was a complementary test to the MIC studied in *S. aureus* and *P. aeruginosa*. The increase of the bacterial population according to observation in real time was evaluated at different concentrations (based on the average PLL content): 500 *μ*M, 250 *μ*M, and 125 *μ*M for *S. aureus* and 200 *μ*M and 125 *μ*M for *P. aeruginosa*. Bacterial counts were performed before incubation (T0) and after 1, 2, 3, 4, 5, and 6 hours of incubation at 37°C. Then, the plate region receiving the lauryl-PLL (25 *μ*L) was resuspended with physiological serum and placed on a Petri dish and incubated at the same temperature for at least 24 hours before confirmation of the potential bacteriostatic or bactericidal effect.

## 3. Results

### 3.1. Characterization of Fatty Acid-PLL

In order to study the linkage between fatty acids and PLL, infrared and gas chromatography techniques were used. The coupling of the fatty acid to PLL was accomplished through the activation of carboxylic acid by ECF. The reaction afforded an amide bond, resulting from the linkage of the acyl chain to the *ε*-lysyl residue of PLL [4, 29–31].

**Figure 2:**
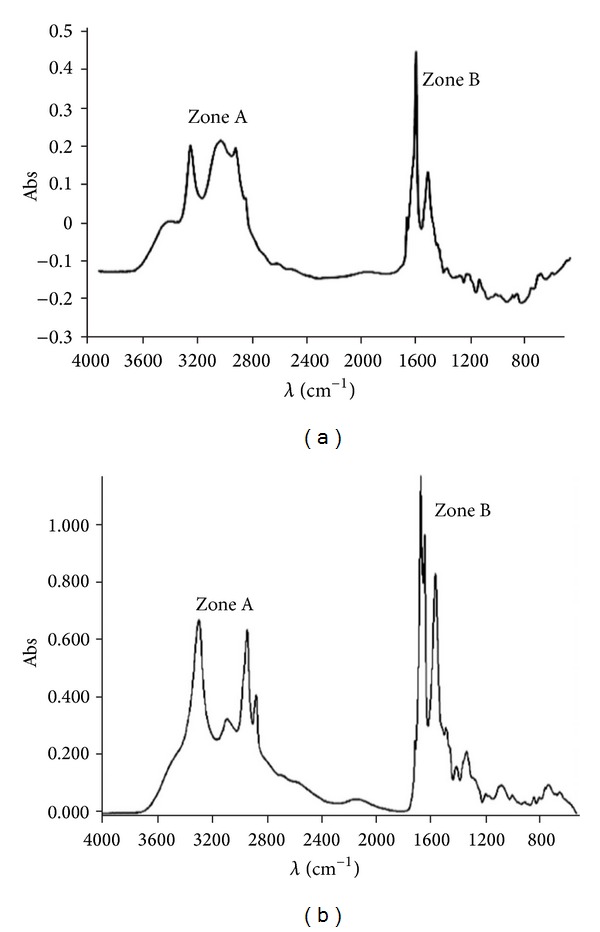
Infrared spectra of poly-L-lysine (a) and lauryl-PLL (b). Fatty acid-PLL compounds were analyzed by ATR-FTIR spectroscopy (with a Bruker apparatus). Zone A: in free PLL (a), 3,200 to 2,850 cm^−1^, CH_2_ groups of side chains of amino acid lysine. In lauryl-PLL (b), CH_3_ groups at the end of the lauric acid chain are not very visible. Note that there is a weak shoulder the CH_2_ peak at 2,925 cm^−1^. Zone B: in free PLL (a), 1,700 to 1,450 cm^−1^, deformation bands of the amino group (2), NH, and of carbonyl groups, CO. In lauryl-PLL (b), these two peaks increase in intensity because of additional CO and NH by the linkage of fatty acid.

In the infrared spectrum of PLL (Figure 2(a)), zone A corresponded to the CH_2_ groups of lysyl residue side chains. Zone B corresponded to the deformation bands of the amino group, NH, and of the carbonyl group, CO. During protein analysis, these two major peaks revealed functions of the bidimensional structure that afforded a random coil structure. Thus, the functions of the folded polypeptide and its sequence of amino acids, the proportions of *α*-helix, *β*-sheet, and loops were evaluated (amine I band). Here, only the results of the infrared spectrum and the concentration of lauryl-PLL are shown. In the infrared spectrum of lauryl-PLL (Figure 2(b)), zone A corresponded to the CH_2_ and CH_3_ groups of the covalently linked lauric acid. A random coil structure was observed. The CH_2_ group of lauric acids involved a shift of 2 cm^−1^ from the reference peak.

**Figure 3:**
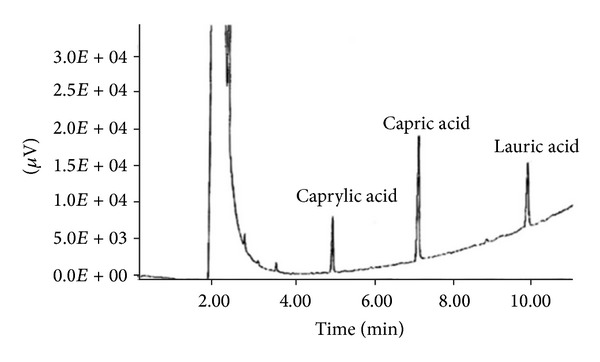
Chromatogram to determine the concentration of lauric acid linked to PLL after acidolysis. The internal standard solution was capric acid (C_10_H_20_O_2_). Using a semicapillary column, the solutes were separated according to the chain-length of the fatty acids. The retention time is proportional to the chain-length of the fatty acids.

Using a gas chromatography technique, we obtained the concentration of lauric acid linked to PLL (Figure 3). Since an internal standard was available (capric acid), the concentration of the linked lauric acid was calculated by comparison of the linked fatty acid with the standard. It was found to be 2 × 10^−3^ M. In general, the concentration of fatty acid linked to PLL varied from 1 × 10^−3^ to 4.5 × 10^−3^ M. Both analytical methods revealed random coupling of fatty acids to PLL.

### 3.2. Evaluation of Fatty Acid-PLL Activity against Bacterial Strains

**Figure 4:**
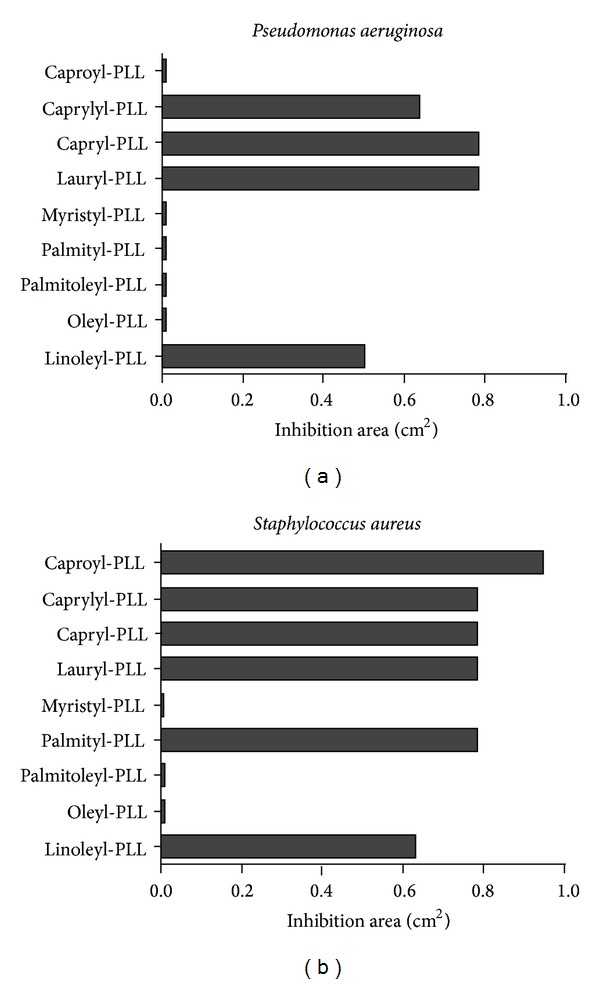
Antibacterial activity of fatty acid-PLL derivatives against *P. aeruginosa* and *S. aureus.* This figure shows the average inhibition area (cm^2^) of the 9 fatty acid-PLL screening at the concentration of grafted fatty acids, 10^−4^ M. The *P. aeruginosa* and *S. aureus* bacterial strains were obtained from clinical isolates.

In these studies, we tested a panel of synthetic fatty acid-PLLs. In order to evaluate the most active products, *in vitro* 9 fatty acid-PLL compounds, C_6_-PLL, C_8_-PLL, C_10_-PLL, C_12_-PLL, C_14_-PLL, C_16_-PLL, C_16:1_-PLL, C_18:1_-PLL, and C_18:2_-PLL, were tested against *S. aureus* (Gram-positive) and *P. aeruginosa* (Gram-negative) in Petri dish assays. The *in vitro* tests were carried out with fatty acid-PLL compounds from the initial concentration of grafted fatty acids at 10^−4^ M, indicating the most effective compounds (Figure 4). For *P. aeruginosa* (Figure 4(a)), C_8_-PLL (caprylyl-PLL), C_10_-PLL (capryl-PLL), C_12_-PLL (lauryl-PLL), and C_18:2_-PLL (linoleyl-PLL) showed antibacterial activity, and for *S. aureus* (Figure 4(b)), C_6_-PLL (caproyl-PLL), C_8_-PLL (caprylyl-PLL), C_10_-PLL (capryl-PLL), C_12_-PLL (lauryl-PLL), C_16_-PLL (palmityl-PLL), and C_18:2_-PLL (linoleyl-PLL) were efficient. These results point to the relationship between antibacterial efficacy and the chain length of the linked fatty acid. In the case of the linoleyl-PLL compound, we observed that a parameter other than the chain length of the fatty acid induced the antibacterial activity.

It was found that 12 carbon units were the optimal acyl chain length linked to PLL, enhancing antibacterial activity. Thus, we performed several studies with the lauryl-PLL compound, which has 12 carbon units.

### 3.3. Determination of the MIC and Kinetic Activity of Lauryl-PLL

As noted for other antimicrobial compounds, the MICs of  lauric acid linked to PLL were 250 *μ*M for *S. aureus*, 125 *μ*M for *P. aeruginosa*, and 1000 *μ*M for *E. coli* (Table 1). These concentrations were compared with conventional antibiotics, including *β*-lactam (benzylpenicillin, oxacillin, ampicillin, amoxicillin, clavulanic acid, ceftazidime, and ticarcillin), aminoglycoside (amikacin), glycopeptide (teicoplanin and vancomycin) antibiotics, and a polypeptide antibiotic (colistin).

The antimicrobial activity of lauryl-PLL with respect to the growth of *S. aureus* and *P. aeruginosa* strains was studied. After two hours, the antibacterial activity against *S. aureus* and *P. aeruginosa* strains reached a maximum at 250 *μ*M and 125 *μ*M, respectively.

### 3.4. Specificity of Lauryl-PLL

The aim in this part of the work was to study the linkage specificity of lauric acid to the *ε*-NH_2_ lysyl of the PLL. First, free PLL, free lauric acid, and a mixture of lauric acid and PLL at the MIC were used as negative controls of the *S. aureus* and *P. aeruginosa* bacterial strains. The final concentrations of each product were those present in the lauryl-PLL compound. These results demonstrated that the linkage of lauric acid to the *ε*-NH_2_ lysyl of the PLL [11, 12] was responsible for the observed antibacterial activity.

Secondly, lauric acid linked to PLO or to PEG performed under the same conditions as the linking of lauric acid on PLL did not show any antibacterial activity against *S. aureus *and* P. aeruginosa* with either free PLO or free PEG and lauryl-PLO or lauryl-PEG.

### 3.5. Screening of Lauryl-PLL against a Broad Variety of Bacteria

**Figure 5:**
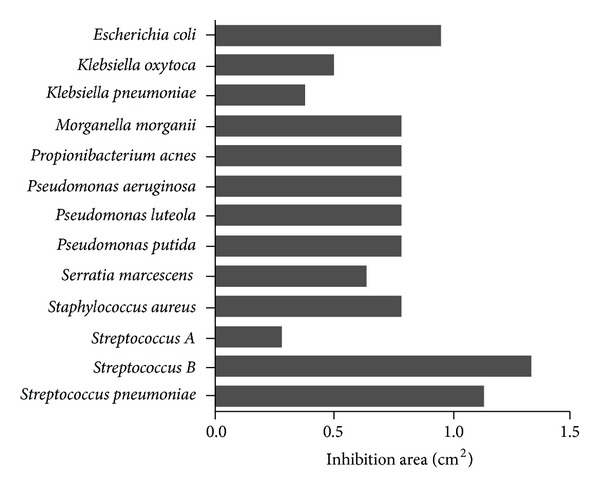
Antibacterial activities of lauryl-PLL against different bacterial strains. The inhibition area (cm^2^) of lauryl-PLL is shown at the concentration of linked lauric acid, 10^−4^ M. 9 Gram-negative and 4 Gram-positive nonresistant strains were tested and revealed the antibacterial effect of lauryl-PLL.

**Figure 6:**
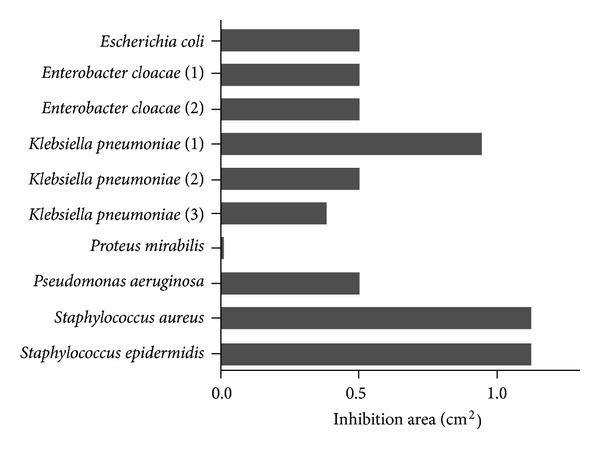
Antibacterial effects of lauryl-PLL against multiresistant bacterial strains. The average inhibition area (cm^2^) of lauryl-PLL is shown at the concentration of linked lauric acid, 10^−4^ M. 5 Gram-negative and 2 Gram-positive multiresistant strains were tested and revealed the antibacterial effect of lauryl-PLL. The number following the name of bacteria (*Enterobacter cloacae* and *Klebsiella pneumoniae*) denotes the number of multiresistant strains tested.

In respect of clinical strains from patients that were multiple-resistant to conventional antibiotics, lauryl-PLL was seen to be active against *Staphylococci* but less so against the multiresistance of *K. pneumoniae* (Figures 5 and 6 and Table 2). However, it was completely ineffective against *Proteus mirabilis*, whether or not it was multiresistant.

## 4. Discussion

The discovery of antimicrobial agents is one of the most important advances in medicine over the past 70 years, alleviating suffering from disease and saving lives. Nevertheless, the development of new agents against resistant bacterial strains is critical. The inappropriate and irrational use of drugs provides favourable conditions for resistant microorganisms to emerge and spread. Antimicrobial resistance is not a new problem but is becoming increasingly more dangerous. Repeated exposure of humans and animals to antibiotics has created what is known as a selection pressure, which tends to favour the mutations and plasmid exchanges responsible for the acquisition of resistance to antibiotics. It also tends to eliminate sensitive bacteria, which are replaced by resistant bacteria. In the case of *S. aureus,* which has been the most frequently isolated bacterial strain in French hospital-acquired infections, it should be noted that more than 90% of the strains were resistant to penicillin [32, 33]. After penicillin-resistance, the ensuing emergence of methicillin-resistant *S. aureus* (MRSA) strains, and since 2002, of vancomycin-resistant strains, has counteracted the success of antibiotherapy [34–36]. Regarding *P. aeruginosa,* this is also pathogen responsible for nosocomial infections in hospitalized patients. Four antibiotic classes, the penicillin/cephalosporins/monobactams, the carbapenems, the aminoglycosides, and the fluoroquinolones, have increased the incidence of acquired resistance by *P. aeruginosa*, called multiresistant *P. aeruginosa *(MRPA). *MRPA* strains have combined several mechanisms of resistance to antibiotics (active efflux, impermeability resulting from the loss of porins, plasmid-encoded enzymes) and additionally are sensitive to colistin (a new cyclic polypeptide) [37–40]. Regarding the *E. coli* strains able to cause severe foodborne disease, these are the most common causes of bladder and kidney infections. A metallo *β*-lactamase, a carbapenemase called NDM-1, has conferred resistance to *β*-lactam antibiotics. Several *Enterobacteriaceae* have developed multiresistance due to NDM-1, inducing resistance to nearly all antimicrobial agents. No therapeutic option exists for infections caused by bacteria producing NDM-1 [41, 42]. Underlying these factors that lead to antimicrobial resistance, depleted arsenal of drugs and vaccines as well as insufficient research and the development of new products must be included. On the one hand, bacterial resistance to a particular drug depends on the mechanism of action of the drug, and, on the other hand, the enzymes encoded by plasmid genes confer natural antibiotic resistance, the bacteria becoming adapted to new environmental circumstances. The resistance to conventional antibiotics is also related to membrane permeability. Thus, the sensitivity of multiresistant bacteria to lauryl-PLL could offer a solution to antibioresistance. The present study continues our research with the cationic PLL compounds and their ability to fight bacterial strains. Here we show that the coupling of the *ε*-amine group of the lysine residue through the acylation of fatty acids increases their antibacterial activity [43, 44]. For the C_6_ to C_18_ fatty acids grafted on PLL, we have developed *in vitro* tests showing that lauryl-PLL (C_12_-PLL) exerts a significant effect at quite low concentrations of covalently linked lauric acid. A change in any of these properties could cause a loss of its antimicrobial activity [45, 46]. The functionality of fatty acid-PLLs is due to the length of the carrier chain, whose molecular mass ranges between 15,000 and 30,000 Da. The linear PLL was used among others as a carrier for drugs or genes [9, 45]. The analytical data suggested that the secondary structure (random coil) of lauryl-PLL acts effectively on the membrane components. It appears that optimal balance must exist among the secondary structure, the hydrophobicity, and the positive charge of PLL compounds in order to increase selective antimicrobial activity [47, 48].

The bactericidal effect of PLL has been approved and authorized by the FDA regarding *ε*-PLL as a food preservative [49, 50]. In contrast, *α*-PLL has been investigated in human gene-delivery systems. This *α*-PLL has several favourable features. It is inert, linear, nonallergic, and non-immunogenic carrier [7, 48]. Furthermore, individual linkage to PLL offers significant advantages, among other aspects, for stabilizing the linked fatty acids. Regarding the hydrophobic part of PLL compounds, the activities of PLL compounds are considered to be more efficacious against Gram-positive than against Gram-negative bacteria [11, 50]. Free saturated fatty acids at 1 mg/mL exert bacteriostatic and bactericidal activity; this activity increases with the length of the chain up to 12 carbons (lauric acid). However, free fatty acids are insoluble [3]. In general, unsaturated fatty acids are usually inactive, and only free linoleic acid is active [12, 51, 52]. In 2006, Kelsey reported that some saturated (capric, lauric, and myristic acid) and polyunsaturated (linoleic acid) fatty acids at concentrations up to 1000 *μ*M may inhibit the growth of *S. aureus* [53]. The free carboxyl group of free fatty acids is necessary for such activity, because ester formation generally decreases the antibacterial effect [54, 55]. Strategies contributing to the reduction of resistant infectious threats include prevention but also new therapeutic weapons, among which are AMPs. Thus, it is essential to gain a better understanding of the molecular mechanism of action of AMPs. Many AMPs, such as *ε*-PLL, are produced at low levels by multicellular organisms, acting on innate immunity. In contrast, *α*-PLL has a much lower antibacterial activity than *ε*-PLL [56]. The main target of many cationic AMPs is the lipid bilayer itself, since these agents do not exhibit any stereospecific interactions with chiral receptors or enzymes [57, 58]. These cationic peptides interact with the negatively charged cell membranes of prokaryotes such as bacteria. Gram-negative bacteria differ from Gram-positive ones in possessing a smaller cell wall peptidoglycan layer between the outer and the inner membranes.The cell wall of Gram-negative bacteria is formed by a peptidoglycan layer, lipopolysaccharides, and porins. Generally speaking, thecell wall plays a size-selective role, in conjunction with secondary protective mechanisms such as active antibiotic efflux and the periplasmic enzyme *β*-lactamase. Gram-negative bacteria are more difficult to treat with new antibacterial agents. Conversely, cationic antimicrobial peptides use a pathway across the cell wall of Gram +/− bacteria [59]. It has been demonstrated that the long-chain fatty acid mechanism of action targets the bacterial cytoplasmic membrane, allowing the entrance of free fatty acids towards the inner membrane [3].Cationic peptides and fragments of proteins are an attractive source of host peptides, since they are less likely to cause antigenic reactions [60]. To date, the mechanism of action of lauryl-PLL remains to be elucidated. Nevertheless, lauryl-PLL, having an amphiphilic character, might show a better ability to adhere and destabilize the bacterial cell wall. PLL with lauric acid, which is more hydrophobic than free PLL, could increase interactions with bacterial membranes. The combination of lauric acid with a polypeptide might shift the balance of electrostatic and hydrophobic forces. Upon increasing the hydrophobicity of fatty acid by PLL, selectivity for bacterial membranes might be decreased [4, 5]. PLL compounds would be electrostatically attracted by the negative groups found on the surfaces of bacteria (lipopolysaccharide and porins in Gram-negative bacteria; lipoteichoic acids and peptidoglycan in Gram-positive species) [56]. Furthermore, lauryl-PLL is reactive against Gram-positive and Gram-negative bacteria, whereas, polymyxins are only used for the treatment of infections caused by Gram-negative bacteria and glycopeptides and are used specifically to fight Gram-positive bacteria. Although electrostatic interaction between positively charged lauryl-PLL and negatively charged phospholipid membranes seems to play an important role in the initial interactions and selectivity, biological activity seems to be driven by the hydrophobic interactions between nonpolar amino acids and the hydrophobic group of the lipid bilayers.

## 5. Conclusions

In sum, here we have tested the effect of an innovative product, lauryl-PLL, on Gram +/− bacteria. Our results suggest that Lauryl-PLL could be a good candidate for the treatment of antibioresistant strains such as *P. aeruginosa*, *E. coli*, and *S. aureus*. Moreover, we observed that lauryl-PLL has an MIC of 125 *μ*M against *P. aeruginosa*, 250 *μ*M against *S. aureus,* and 1000 *μ*M against *E. coli*, which are very low effective concentrations. Finally, the treatment of these strains with a very low quantity of product is devoid of undesirable side effects.

## Figures and Tables

**Table 1:** Comparison of MIC between lauryl-PLL and conventional antibiotics.

Bacterial strains	Lauryl-PLL (*μ*M of linked lauric acid)	Sensitivity and name of conventional antibiotics (*μ*M)
*Staphylococcus aureus *	250	Oxacillin: 1.25 (R)
		Benzylpenicillin: 0.75 (R)
		Vancomycin: ≤0.699 (S)
		Teicoplanin: ≤0.32 (S)

*Escherichia coli *	1000	Ampicillin: ≥91.6 (R)
		Amikacin: 13.66 (S)
		Amoxicillin: 10.95 (S)
		Clavulanic acid: 20.08 (S)

*Pseudomonas aeruginosa *	125	Ticarcillin: ≥333 (R)
		Ceftazidime: ≥117 (R)
		Amikacin: 27 (I)
		Colistin: 1.7 (S)

A letter following the name of the antibiotics refers to the sensitivity to antibiotics: R: resistant; I: intermediate; S: sensitive. Based on the antibiograms of the bacterial strains tested, we selected some known and widely used conventional antibiotics, as well as polypeptide and glycopeptide antibiotics with activity similar to that with lauryl-PLL. Their sensitivity and their MICs were tested in nonresistant strains: *S. aureus* (Gram-positive) and *P. aeruginosa*, and *E. coli* (Gram-negative).

**Table 2:** Description of multiresistant bacteria.

Multiresistant bacteria	Resistance to antibiotics
*Escherichia coli *(*N* = 7/19)	Ampicillin, cefalotin, ciprofloxacin, nalidixic acid, norfloxacin, ofloxacin, ticarcillin

*Enterobacter cloacae *(1) (*N* = 15/19)	Amoxicillin/clavulanic acid, ampicillin, cefalotin, cefotaxime, cefoxitin, ceftazidime, ciprofloxacin, gentamicin, nalidixic acid, netilmicin, nitrofurantoin, norfloxacin, ofloxacin, ticarcillin, tobramycin

*Enterobacter cloacae* (2) (*N* = 6/19)	Amoxicillin/clavulanic acid, ampicillin, cefalotin, cefotaxime, nalidixic acid, norfloxacin

*Klebsiella pneumoniae* (1) (*N* = 8/19)	Ampicillin, ciprofloxacin, gentamicin, nalidixic acid, nitrofurantoin, norfloxacin, ofloxacin, ticarcillin

*Klebsiella pneumoniae* (2) (*N* = 14/19)	Amoxicillin/clavulanic acid, ampicillin, cefalotin, cefotaxime, ceftazidime, ciprofloxacin, nalidixic acid, netilmicin, nitrofurantoin NCCLS, norfloxacin, ofloxacin, ticarcillin, tobramycin, trimeth-sulfamethoxazole

*Klebsiella pneumoniae* (3) (*N* = 17/19)	Amikacin, amoxicillin/clavulanic acid, ampicillin, cefalotin, cefotaxime, ceftazidime, ciprofloxacin, gentamicin, nalidixic acid, netilmicin, nitrofurantoin, norfloxacin, ofloxacin, piperacillin/tazobactam, ticarcillin, tobramycin, trimethoprim/sulfamethoxazole

*Pseudomonas aeruginosa *(*N* = 13/18)	Aztreonam, cefepime, ceftazidime, ciprofloxacin, imipenem, meropenem, minocycline, pefloxacin, piperacillin, piperacillin/tazobactam, ticarcillin, ticarcillin/clavulanic acid, trimethoprim/sulfamethoxazole

*Staphylococcus aureus *(*N* = 4/22)	Benzylpenicillin, kanamycin, oxacillin, tobramycin

*Staphylococcus epidermidis *(*N* = 9/22)	Benzylpenicillin, erythromycin, gentamicin, kanamycin, lincomycin, oxacillin, pristinamycin, quinupristin, tobramycin

This table completes the information shown in Figure 6. The letter, *N*, corresponds to the number of resistant antibiotics in comparison with the total number of antibiotics tested against the bacterial strains. The inhibition area (cm^2^) of lauryl-PLL is shown at the concentration of grafted lauric acid, 10^−4^ M.
